# The International Pharmacists-as-Immunizers Partnership (IPIP)—Advancing Research on Pharmacist-Administered Immunizations Worldwide

**DOI:** 10.3390/pharmacy7020053

**Published:** 2019-06-04

**Authors:** Richard R. Violette, Sherilyn K. D. Houle, Lisa M. Nissen, Nancy M. Waite

**Affiliations:** 1 School of Pharmacy, University of Waterloo, Waterloo, ON N2G 1C5, Canada; rviolett@uwaterloo.ca (R.R.V.); nmwaite@uwaterloo.ca (N.M.W.); 2 International Pharmacists-as-Immunizers Partnership (IPIP), University of Waterloo, Waterloo, ON N2G 1C5, Canada; 3 Faculty of Health, School of Clinical Sciences, Queensland University of Technology, Brisbane, QLD 4000, Australia; l.nissen@qut.edu.au

**Keywords:** pharmacists, immunization, research, network, international

## Abstract

This article describes the formation of the International Pharmacists-as-Immunizers Partnership (IPIP), an international network of pharmacy practice researchers with an interest in pharmacist-administered immunizations. Using funds obtained from a university-sponsored grant, a two-day meeting was held at the University of Waterloo in Canada to discuss published and in-progress research on the topic, identify gaps and priorities for future research, and share implementation strategies used in different jurisdictions. Twelve researchers from five countries attended this initial meeting, identified from both personal networks and from authorship lists from published research. Small- and large-group discussions addressed a number of themes, including: clinical, economic and educational outcomes of the service; the perspectives of pharmacists, patients, and other health professionals; operational and policy factors influencing uptake; safety; and the immunizing pharmacist’s role in disaster preparedness. Feedback on our first meeting and outcomes achieved were evaluated on the basis of participant feedback. Key components of the meeting that were considered successful and important lessons learned are summarized, so that other like-minded researchers with a shared pharmacy practice research interest could consider leveraging funding opportunities to establish other international pharmacy practice research networks.

## 1. Introduction

Pharmacists worldwide are increasingly being granted the authorization to administer vaccines and other drugs and, in some cases, to receive publicly funded remuneration for this service [1,2]. First introduced in the United States nearly 25 years ago [3], pharmacist-administered immunizations have since been adopted across all 50 states in the USA, as well as in a number of other countries (Argentina, Australia, Canada, Costa Rica, Denmark, France, Ireland, Italy, New Zealand, Philippines, Portugal, South Africa, Switzerland, United Kingdom, and others) [4] and also across a broadening range of vaccine and drug products [5,6]. While published evidence is still scarce, it is in fact within scope to administer any drug in some jurisdictions including a number of provinces in Canada [7].

Research on the impact of pharmacist-administered immunizations to date has demonstrated high patient satisfaction (with particular appreciation for the convenience and accessibility of community pharmacists as vaccination providers) [8,9] as well as improved vaccination rates [10]. However, a number of limitations in the current body of research remain:

- Much of the research on patient outcomes and impact on immunization rates has been conducted in the United States;
- There has been some unintentional duplication of work done in different jurisdictions;
- Some areas of research have not been adequately addressed (for example, quality improvement and implementation science);
- The identification of similar findings regarding gaps in care (for example, service delivery to vulnerable populations) and pharmacy operational challenges related to implementation of these services. This suggests that a coordinated strategy to conduct research in this area may be more efficient and effective in creating evidence to address research, practice and policy questions.

## 2. Formation of the Partnership

The International Pharmacists-as-Immunizers Partnership, IPIP, was initiated through an International Research Partnership Grant program offered by the University of Waterloo, Ontario, Canada. These are “internal seed grants that provide Waterloo researchers with incentives to develop new or existing international research collaborations with leading institutions known for high-quality research” across all disciplines of research [11]. This funding must be matched by an international partner, and Queensland University of Technology (Brisbane, Australia) was successful in obtaining funding. The joint proposal was to host the first known international gathering of pharmacy practice researchers to tackle the global implications of pharmacists-as-immunizers research. The goals of this meeting were broadly defined to:

- Identify existing research gaps;
- Generate research capacity;
- Foster collaboration across international jurisdictions;
- Develop competitive funding applications to address research gaps;
- Develop engagement and knowledge translation strategies for the uptake of research into policy and practice change.

Invitations to participate were sent to a group of pharmacy practice researchers and practicing pharmacists representing various geographic areas and research interests, seeking their commitment to attend an initial meeting, to share their existing research and practice experience, and to participate in the generation of new knowledge related to pharmacists as immunizers. These individuals were identified from existing networks, as well as by identifying those who authored published research in the area. In preparation for the initial meeting, a literature search of published research was performed in a systematic manner with medical librarian assistance, and participants were asked to submit additional examples of their completed and in-progress research projects. These were compiled into an evidence brief that summarized this work, with the goal of identifying gaps and areas of saturation. This report was shared with meeting attendees in advance to help guide discussion and ensure all participants were aware of existing research.

**Figure 1:**
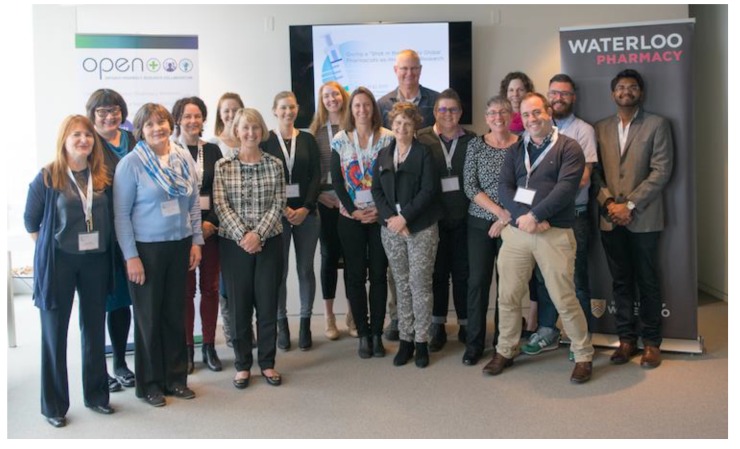
International Pharmacists-as-Immunizers Partnership (IPIP) meeting participants. Nancy Waite, Claire Anderson, Mary Hayney, Natalie Gauld, Sherilyn Houle, Jean-Venable “Kelly” R Goode, Kaitlyn Porter, Libby McCourt, Jean Spinks, Gregory Kyle, Beverley Glass, Lisa Nissen, Fiona Kyle, Jennifer Isenor, Chris Campbell, Richard Violette, Gokul Raj Pullagura.

Twelve researchers and four graduate students from five countries attended a two-day meeting hosted at the School of Pharmacy, University of Waterloo, in April 2017 (Figure 1). The first day’s activities started with a country-by-country review of pharmacists-as-immunizers current policy, immunization challenges and key drivers for increased participation of pharmacists in this space. This was followed by roundtable discussions, informed by the evidence brief, with the purpose of identifying research gaps (see Table 1), priorities, and strategic directions for future research, including identifying potential collaborators, funding sources, and partnerships with stakeholders and knowledge users. Discussions were guided by addressing the following broad themes:

- Impact on outcomes;
- Pharmacist perspectives;
- Patient/public perspective;
- Other healthcare professional perspectives;
- Pharmacy operational factors;
- Legislative/policy factors;
- Economic outcomes;
- Patient demographics;
- Emergency/disaster preparedness;
- Educational outcomes;
- Pharmacist/patient safety.

The second day consisted of a series of working group sessions to outline project proposals and to develop a strategic plan and timeline for follow-up work. Approximately 18 months after the meeting, the participants were contacted by the meeting organizers to provide an update on their work associated with IPIP and to provide feedback on the process of forming the network.

## 3. Outputs and Future Directions

The literature compiled to form the pre-meeting Evidence Brief will be written up in the form of a scoping review to facilitate the sharing of this information with others interested in pharmacists-as-immunizers research. A jurisdictional review describing the scope of pharmacist-administered immunizations worldwide is currently under review for publication. In addition, a number of commentary and related research brief articles will share lessons learned by jurisdictions who have introduced and modified this service, so that others looking to introduce or expand their immunization services can benefit from this experience. Following the completion of these foundational pieces, the IPIP team members plan to re-convene to identify areas of priority and draft research proposals for funding to address some of the remaining research gaps. In addition to research outputs, the formation of the partnership has facilitated international visits of post-doctoral trainees and graduate students across institutions, informal meetings held at international conferences, and peer reviews of grant applications and research manuscripts.

## 4. Lessons Learned and Potential for Expansion to Other Areas of Research

Pharmacy practice is evolving worldwide, yet many researchers and policy-makers work within geographically defined silos. This potentially results in unnecessary duplication of research, gaps in creating robust evidence needed to advance pharmacy practice, inefficient practice roll-out from not sharing and replicating what was successfully done elsewhere, and small-scale research trials whose results may lack generalizability. Indeed, many IPIP participants appreciated the opportunity to meet researchers with similar interests and a commitment to collaboration. Other lessons learned from the experience are summarized below:

- There were more differences in policy and implementation of pharmacists-as-immunizer services than expected across jurisdictions. Much of the discussion among participants was related to understanding these differences and learning from the unique experiences of others;
- While the researchers may have known of some of the others invited, in general, they had not met in person before and had never had the opportunity for concentrated research discussions on this topic;
- Realistic expectations need to be set for what can be accomplished in two days. While the meeting goal was to conclude with the generation of concrete international grant ideas, the sharing of experiences and research was prioritized at this early stage of network formation;
- While the focus of the workshop was pharmacists as immunizers, the interconnectedness of various pharmacist activities could not be ignored. Discussion often expanded into other pharmacist services and regulation, such as authority to prescribe (as it pertained to vaccines but then further into a broader discussion of prescribing authority), generating additional potential research topics for collaboration;
- While time was limited for social connections, a dinner, ice-breakers, and assigning participants to small groups with an intention to balance personalities, research interests, home country, and perspectives were all very important to consider and, in this case, led to positive and lasting connections;
- Having funding for the event and being able to cover travel, accommodation, and food costs was critical to ensure participant attendance in person;
- Preparation of the evidence brief prior to the meeting (including the incorporation of current research being conducted by the researchers) was labor-intensive but was well worth the effort to move the discussion forward more efficiently;
- Maintaining momentum for collaborative outputs post-meeting was a challenge, which was further compounded by geography. Firm commitments, clear expectations on deliverables, and a timely follow-up meeting would all be important facilitators to ensure continued productivity.

Leveraging funding from a university-provided grant, the formation of IPIP provides an example of the establishment of a formalized international network of researchers with similar interests to support ongoing collaboration. Our primary aim in this paper was to describe how funding can be leveraged to form international collaborations, yet also to highlight some of the challenges and lessons learned along the way. Networks of this nature can complement efforts by international pharmacy associations to support practice-based research and provide both junior and established researchers with an expanded network of individuals who may be willing to collaborate on cross-jurisdictional research. Pharmacy practice researchers are encouraged to consider pursuing similar collaborations in their area of interest, seeking funding from universities, government and non-government programs, industry, or not-for-profit organizations to establish networks that support international collaboration with the shared goal of improving patient care through quality pharmaceutical care.

## Figures and Tables

**Table 1:** Examples of research gaps identified.

Theme	Example of Research Gap Identified
Impact on outcomes	Impact on hard-to-reach, high-risk, and vulnerable groups
Pharmacist perspectives	Operational challenges to vaccination
Patient/public perspectives	Vaccine hesitancy in community pharmacy
Other healthcare professional perspectives	Strategies to increase inter-professional collaboration
Pharmacy operational factors	Task shifting to registered pharmacy technicians
Legislative/policy factors	Lessons learned and strategies identified from policy change across different jurisdictions
Economic outcomes	Immediate and long-term economic outcomes of pharmacists-as-immunizers
Emergency/disaster preparedness	Linking universities and pharmacies with the public through a disaster preparedness plan
Educational outcomes	Differences in national competencies and accreditation requirements
